# Comparative Genomics and Phenotypic Investigations Into Antibiotic, Heavy Metal, and Disinfectant Susceptibilities of *Salmonella enterica* Strains Isolated in Australia

**DOI:** 10.3389/fmicb.2019.01620

**Published:** 2019-07-16

**Authors:** Annaleise Wilson, Edward M. Fox, Narelle Fegan, D. Ípek Kurtböke

**Affiliations:** ^1^Genecology Research Centre and the School of Science and Engineering, University of the Sunshine Coast, Maroochydore, QLD, Australia; ^2^Food Safety and Stability Group, Agriculture and Food, CSIRO, Werribee, VIC, Australia; ^3^Department of Applied Sciences, Northumbria University, Newcastle upon Tyne, United Kingdom

**Keywords:** *Salmonella enterica*, antibiotic resistance, heavy metals, Australia, next generation sequencing, *bla*_*TEM–1*_, *tetA*, biocides

## Abstract

*Salmonella enterica* is recognized as a major contributor of gastrointestinal illness worldwide. Concerns have been raised over the increasing prevalence of antibiotic resistant strains of *Salmonella* isolated from animals and food, and the role of antibiotics and other antimicrobial agents such as biocides and heavy metals in the selection and dissemination of antibiotic resistant bacteria to human hosts. In this study the antibiotic, heavy metal and disinfectant resistance genotypes and phenotypes of 19 *S. enterica* isolates from food-producing animals were established using whole genome sequence analysis, disc diffusion, as well as broth or agar dilution methods. This study also investigated the genomic environment of resistance genes on mobile genetic elements and chromosomal DNA. An ampicillin and streptomycin resistant *S.* Infantis isolate in this study harbored a β-lactamase (*bla*_*TEM–1*_), and two streptomycin resistance conferring genes (*strA* and *strB*) on a class 1 integron mobilized on a large conjugative plasmid. This plasmid also harbored two arsenic resistance gene cassettes. The arsenic resistance cassette, *arsRCDAB*, was also observed in two *S.* Singapore isolates with high tolerance to arsenate. A nalidixic acid resistant *S.* Typhimurium isolate was found to possess a mutation in *gyrA* resulting in amino acid change Asp87Gly and tetracycline resistant *S.* Typhimurium isolate was found to harbor efflux pump gene, *tetA*. No resistance (genotypic or phenotypic) was recorded to the disinfectants screened in this study. Taken together, results of this study showed a good correlation between predicted and measured resistances when comparing genotypic and phenotypic data, respectively. The findings of this study do not suggest resistance to clinically relevant antibiotics are widespread among *Salmonella* isolated from Australian food-producing animals.

## Introduction

*Salmonella enterica* is a Gram-negative bacterial pathogen that is the cause of the foodborne illness, salmonellosis (Coburn et al., 2007). In 2011, non-typhoidal *Salmonella* spp. caused approximately 12,271 reported cases of gastroenteritis in Australia, the second highest causative agent of this disease after *Campylobacter* spp. (The OzFoodNet Working Group, 2011). Additionally, the incidences of salmonellosis in Australia has increased significantly in the past decade (Ford et al., 2016). *Salmonella* is most frequently transmitted through contaminated food products or by the handling of infected animals or feces in the farm environment (Hundy and Cameron, 2002; Pesciaroli et al., 2017). Outbreaks of salmonellosis are often attributed to the consumption of contaminated eggs or chicken but have also been reportedly attributed to contaminated pork, beef, and fresh produce (Delpech et al., 1998; Combs et al., 2006; Munnoch et al., 2009; Friesema et al., 2012; Moffatt et al., 2016). The food production and supply chain plays a critical role in the spread of *Salmonella* by connecting its environmental niches, animal and human hosts and consequently has been the focus of research and intervention strategies to reduce the bacterial load in food. Recently, the Australian and New Zealand Ministerial Forum on Food Regulation developed the “Australia’s Foodborne Illness Reduction Strategy 2018 – 2021+”^1^ with the vision of reducing the number of human cases of illness caused by *Salmonella* and *Campylobacter* (Australian Department of Health, 2018). A key feature of this strategy is the support of research and development on the epidemiology of salmonellosis and strategies for its prevention.

Antimicrobial resistance in *Salmonella* spp., including resistance to antibiotics, heavy metals and sanitizers, is a significant concern due to its relevance to human health (WHO, 2001). Antibiotics play an important role in the treatment of invasive salmonellosis and in gastrointestinal disease in immunocompromised patients. Despite increased antibiotic stewardship in Australia, antimicrobials are widely used as a treatment for animal infections, as growth promotors or for prophylactic uses (Mayrhofer et al., 2006; Lean et al., 2013). In modern food production disinfectants/sanitizers are used for controlling bacterial load in food processing facilities and on associated equipment. Antibiotic resistance (AMR) and multidrug resistance (MDR) in *Salmonella* spp. isolated in Australia has increased in recent decades, with MDR prevalence increasing from 3.4% in 1984 to 9.7% in 2013 (Williamson et al., 2018). Additionally, it has been suggested that exposure and adaptation to particular biocides, as well as heavy metals, could also lower the susceptibility of cells to some antibiotic compounds, a phenomenon called cross-resistance (Copitch et al., 2010; Condell et al., 2012). Disinfectant resistant strains of *Salmonella* have also been identified in food production facilities and retail foods presenting a significant public health concern (Long et al., 2016; Deng et al., 2017). Microflora and pathogens may also be exposed to non-essential heavy metals, such as cadmium, chromium, arsenic and mercury as a result of environmental pollution. Industrial processing and fertilizer use can lead to an accumulation of heavy compounds in the soil, air and water associated with food production facilities and the farming environment (Liu et al., 2015; Li et al., 2017). As cellular mechanisms employed by cells to expel heavy metals are similar to others responsible for antibiotic resistance (i.e., efflux pumps), resistance to heavy metals may also result in reduced susceptibility to clinically relevant antibiotics (Yu et al., 2017). Additionally, micronutrients such as copper and zinc are added to animal feed lots to increase feed efficiency and promote animal growth (Medardus et al., 2014). High concentrations of these elements can be cytotoxic to bacteria, and these compounds can upregulate the expression of stress response proteins (Lim et al., 2002). Despite this trend, only a small number of studies have analyzed underlying genetic mechanisms of disinfectant or heavy metal resistance among *Salmonella* strains isolated in Australia. Additionally, few studies have analyzed the genomic context of these mechanisms on mobile genetic elements such as plasmids, bacteriophage and transposons that can be disseminated to other bacterial cells via horizontal gene (Ghosh et al., 2000; Butaye et al., 2003; Bennett, 2008; Zhang et al., 2017).

Detection of resistance genotypes and mobile genetic elements (MGEs) has traditionally been conducted using PCR amplification with established primers or through DNA microarray analysis. However, these techniques have less resolution when compared to next generation sequencing technologies. Advances in whole genome sequencing (WGS) approaches and reduced costs have made WGS a viable alternative to conventional methods when used for “resistome” and/or “mobilome” identification and characterization (Alam et al., 2014; den Bakker et al., 2014; Tyson et al., 2015). WGS adds higher resolution to AMR investigation by allowing researchers to view the context in which AMR genes reside within the bacterial genome, for example on MGEs (Wilson et al., 2018). Given the importance of AMR and the role of MGEs in its dissemination, the aim of this study was to: (i) utilize phenotypic screening of AMR in *Salmonella* isolated in Australia to characterize resistance to antibiotics and biocides; (ii) utilize WGS and comparative genomics analyses to characterize the resistance genes present among a cohort of *Salmonella* isolated in Australia, and examine the location of these genes within the bacterial genome; (iii) characterize mobile genetic elements among the isolate cohort; (iv) examine the utility of WGS for predicting AMR phenotypes in *Salmonella* isolates. The presented study thus used traditional phenotypic susceptibility testing to determine any discrepancies between AMR genotype profile and phenotypic profiles. Findings of this study may assist in developing insights into the cellular mechanisms that are related to (or underlie) the pathogen’s survival in the presence of toxic compounds and may inform strategies to control *Salmonella* in food products and prevent human illness.

## Materials and Methods

### *Salmonella* Isolates Analyzed in This Study

This study evaluated 19 *S. enterica* strains isolated between 2001 and 2013 from Australian food production chains (Table 1). Sampling and confirmation of isolates has been previously characterized (Duffy et al., 2009, 2010). All isolates were sent to either the Microbiological Diagnostic Unit (University of Melbourne, Melbourne, VIC, Australia) or Queensland Health Scientific Services (Coopers Plains, Brisbane, QLD, Australia) for serotyping and phage−typing. Isolates represented serotypes of significance to public health, including *S.* Typhimurium, as well as *S.* Infantis and *S.* Singapore, which are highly prevalent within the poultry industry in Australia (Cox et al., 2002). In the case of *S.* Typhimurium, Phage Types 44, 135/135a, 108/9, and 170 were included as they are important to human illness in Australia (The OzFoodNet Working Group, 2010, 2011; Moffatt et al., 2017). Strains were grown on tryptic soya agar (TSA; Oxoid) for 24 h at 37°C before transfer in tryptic soy broth (Oxoid) containing 20% glycerol (v/v) for long-term storage at −80°C. Isolates were subcultured onto TSA with 16 ± 2 h incubation at 37°C prior to *in vitro* experimentation and DNA extraction.

**TABLE 1:** *Salmonella enterica* isolates included in this study and their metadata.

**Bacterial strains**	**Phage type**	**Isolation date**	**Source**
**S. Typhimurium**			
Sal-12	44	November 19, 2001	Goat feces
Sal-43	135	November 19, 2001	Goat feces
Sal-240	170	November 20, 2001	Goat feces
Sal-1000	135	November 27, 2002	Cattle feces
Sal-1003	170	December 9, 2002	Cattle feces
Sal-1013	9	January 8, 2003	Cattle feces
Sal-1576	135a	July 14, 2005	Chicken carcass
Sal-1657	135	December 7, 2006	Dairy factory
Sal-1705	135	February 27, 2007	Sheep fleece
Sal-2103	135a	October 8, 2007	Chicken carcass
Sal-2323	9	May 16, 2008	Chicken carcass
Sal-2327	135a	May 16, 2008	Chicken carcass
Sal-2355	135	May 22, 2009	Chicken carcass
Sal-2413	108	March 28, 2013	Cattle feces
**S. Infantis**			
Sal-576		February 26, 2002	Goat feces
Sal-1023		March 4, 2003	Cattle feces
Sal-1457		May 31, 2005	Chicken carcass
**S. Singapore**			
Sal-1234		April 18, 2005	Chicken carcass
Sal-2350		May 21, 2009	Chicken carcass

### Genome Sequencing, Assembly, and Phylogenic Analysis

Genomic DNA extractions were performed using the DNeasy Blood and Tissue Kit, as per the manufacturer’s instructions (Qiagen, United Kingdom). DNA quality (including measurements of protein, carbohydrate and phenol contamination) and concentration (ng/μl) was determined using a NanoDrop ND1000^TM^ (Thermo Fisher Scientific, Australia) and agarose gel electrophoresis. DNA was standardized to 5 ng/μl before storage at −20°C. The Nextera XT Library Preparation Kit (Illumina, United States) was used, and sequencing was performed using the Illumina MiSeq platform (Illumina) by the Ramaciotti Centre for Genomics (UNSW Sydney, Australia).

Prior to assembly, FASTQ files were evaluated for quality using FastQC v0.70 (Andrews, 2010). Sequencing reads were trimmed using Trimmomatic v0.33 (Bolger et al., 2014) to remove Illumina adapters and low quality reads. The application was run on settings phred33, sliding window size of 4 bp and average quality requirement of lower than Q15. *De novo* assembly of trimmed paired-end libraries was performed using the open source platform Species Prediction and Diversity Estimation (SPAdes) v 3.11 (Bankevich et al., 2012) with specified k-mer sizes of 21, 33, 55, and 77 to create multiple contiguous sequences (FASTA files). Contiguous sequences of less than 200 bp or with a coverage value less of than 5× were then removed from the dataset. The FASTA files were processed through the online annotation platform Rapid Annotation using Subsystem Technology (RAST), to generate GENBANK files (Aziz et al., 2008; Overbeek et al., 2014; Brettin et al., 2015). GENBANK files were visualized and analyzed using Geneious v10.2.3 software (Biomatters Limited). Circular BLAST comparison of draft whole genome sequences was performed using an in-house instance of BLAST Ring Image Generator (BRIG) v0.95 (Alikhan et al., 2011).

Variant calling of SNPs and insertions/deletions in *S.* Typhimurium stains was performed using SNIPPY v2.5^2^, by mapping the DNA sequence reads with the *S.* Typhimurium LT2 reference genome (Accession Number: AE006468). SNPs identified as phage, plasmid and repeat regions were removed from the dataset. Phylogenic analysis of *S. enterica* isolates was performed using SplitsTree4 (Huson, 1998) using the NeighborNet algorithm (Bryant and Moulton, 2004).

All genomes were submitted to NCBI under the following Accession Numbers: RQPK00000000, RQPL00000000, RQPM00000000, RQPN00000000, RQPO00000000, RQPP00000000, RQPQ00000000, RQPR00000000, RQPS00000000, RQPT00000000, RQPU00000000, RQPV00000000, RQPW00000000, RQPX00000000, RQPY00000000, RQPZ00000000, RQQA00000000, RQQB00000000, and RQQC00000000.

### Identification and Classification of Mobile Genetic Elements

Plasmids were detected and typed using PlasmidFinder 2.0, a web tool designed to identify plasmids in Enterobacteriaceae sequence reads (Carattoli et al., 2014). Plasmid regions were manually extracted in Geneious as GenBank files and visualized and compared using EasyFig v2.2.3 (Sullivan et al., 2011). Prophage insertions were identified using the online platform PHASTER (Zhou et al., 2011; Arndt et al., 2016). Phage regions were extracted and analyzed in NCBI nucleotide BLASTn service to confirm their identity. Transposase and insertion sequence classification was performed using ISFinder (Siguier et al., 2006).

### Identification of Antibiotic, Heavy Metal, and Disinfectant Resistance (AMR) Determinants

Identification of acquired antimicrobial resistance genes and chromosomal point mutations that confer antimicrobial resistance phenotypes was performed using Resistance Gene Identifier (RGI), a resistome prediction tool which uses BLAST algorithms using curated AMR genes and SNPs available in the Comprehensive Antibiotic Resistance Database (Jia et al., 2017). A secondary enquiry was performed using the webserver ResFinder 3.4^3^. The databases include AMR determinants for β-lactams, fluoroquinolones, aminoglycosides, tetracyclines, phenicols, trimethoprim, and sulphonamides. Detection of mutations in the Quinolone Resistance Determining Regions of DNA gyrase genes *gyrA* and *gyrB* and DNA topoisomerase IV genes *parC* and *parE* was performed through alignment with reference genes downloaded from the National Centre for Biotechnology Information. The reference genes were pairwise aligned to the corresponding genes annotated from the isolate’s draft genome sequence using the ClustalW alignment tool (Thompson et al., 1994).

Identification of biocide and heavy metal resistance genes was performed using the BLASTn search engine available through BacMet^4^ a curated database of predicted and experimentally confirmed resistance genes (Pal et al., 2014). Text-based queries of sequence annotations was also used to search for antimicrobial resistance genes using Geneious v10.2.3 software package (^5^ Biomatters Limited).

### Determination of Antibiotic Susceptibility Phenotype

The sensitivity of all 19 isolates in this study to a panel of 14 antibiotics was determined by disc diffusion performed in accordance with the Clinical Laboratory Standards Institute (CLSI) standards (Clinical and Laboratory Standards Institute, 2017). The antibiotic panel included for testing comprised of ampicillin (10 μg), streptomycin (10 μg), tetracycline (30 μg), cefoxitin (30 μg), sulphafurazole (100 μg), florfenicol (30 μg), norfloxacin (10 μg), kanamycin (30 μg), gentamicin (256 μg), ciprofloxacin (5 μg), chloramphenicol (30 μg), trimethoprim-sulfamethoxazole (23.75 μg), and nalidixic acid (30 μg). An *Escherichia coli* strain (ATCC^®^ 25922^TM^) was used as quality control (QC) to achieve the minimum performance standards as dictated by the CLSI (Clinical and Laboratory Standards Institute, 2017). This assay was performed in duplicate on separate occasions.

### Determination of Heavy Metal Susceptibility Phenotype

The isolates were subjected to heavy metal susceptibility testing by the agar dilution modified method to that previously described. Sensitivity was measured against six heavy metals: arsenic (NaAsO_2_; 0–20 mM), cadmium (CdCl_2_; 0–20 mM), cobalt (CoCl_2_; 0–20 mM), copper (CuSO_4_; 0–32 mM), lead [Pb(NO_3_)_2_; 0–15 mM], and zinc (ZnSO_4_; 0–20 mM). Cultures were grown for 16 ± 2 h on TSA plates at 37°C. Three to five colonies were then inoculated into 3 ml of Maximum Recovery Diluent (Oxoid) to make a solution with a turbidity equal to that of a 0.5 McFarland standard. A 20 μl aliquot of the bacterial suspension was transferred dropwise onto the surface of the Isosensitest agar (Oxoid) containing two-fold increases in heavy metal concentrations. The plates were allowed to dry before being incubated for 24 h at 37°C. Following incubation, the plates were observed for the presence of bacterial growth. The minimum inhibitory concentration (MIC) was interpreted as the minimum concentration of heavy metal that inhibited the growth of the bacterium. A sterile loop was used to scrape the surface of the agar plate where the bacteria culture was dropped to harvest any remaining viable cells. The cells were streaked across the surface of a TSA plate void of heavy metals and incubated for a further 24 h at 37°C. The minimum bactericidal concentration (MBC) was determined to be the lowest concentration of heavy metals where no growth occurred after re-culturing on TSA. This assay was performed in duplicate on separate occasions.

### Determination of Disinfectant Susceptibility Phenotype

The isolate’s susceptibility profile was tested against three disinfectants commonly used in the food production facilities. Benzalkonium chloride (QAC; 0.5–256 μg/ml), chlorhexidine gluconate (cation; 0.06–64 μg/ml), and triclosan (phenol; 0.125–1024 μg/ml). The MIC and MBC for each disinfectant was determined by using a modified broth microdilution method to that previously described (Condell et al., 2012). Isolates were streaked on TSA and incubated at 37°C for 16 ± 2 h. A colony of each isolate was suspended in 10 ml of Mueller Hinton broth (MHB) and incubated at 37°C until late logarithmic phage. A 100 μl aliquot of the incubated culture was added to 9.9 ml of MHB to form a 1:10,000 dilution containing approximately 10^5^ log_10_ CFU/ml. A 100 μl aliquot of the bacterial culture was then transferred into wells of 96-well microtiter test plate containing 100 μl of MHB containing 2-fold concentrations of the disinfectants/sanitizers. The microtiter plate was incubated for 24 h at 37°C. The minimum inhibitory concentration was assessed by turbidimetric analysis. Bacterial growth was determined using a spectrophotometer with a wavelength of 600 nm, recorded every 30 min over a 24 h period. To establish the background OD, readings taken at 0 m, 30 m, and 1 h were averaged. If the OD of the experimental wells exceeded the background OD, the well was considered positive for bacterial growth. The MIC was designated as the lowest biocide concentration at which the OD of the test well did not exceed the optical density of the background OD. Following the 24 h test period, a 100 μl aliquot of each well was transferred to 100 μl of Dey-Engley Neutralizing Broth (Sigma-Aldrich, St. Louis, MO, United States) and allowed to incubate for 2 minutes at room temperature. A 100 μl aliquot was then spread onto the surface of a MH agar plate and incubated at 37°C for 18–24 h (Skowron et al., 2018). The MBC was designated as the lowest biocide concentration which resulted in no viable growth of that isolate. This assay was performed in duplicate on separate occasions.

### Ethics Statement

No ethical approval was obtained as this study did not involve animal or human subjects. The study used existing isolates from the CSIRO culture collection. New samples or isolates were not collected as part of the study and based on this no ethics approval was required for this study as defined by University of the Sunshine Coast’s research ethics arrangements and the National Statement on Ethical Conduct in Human Research. CSIRO own these samples and gave permission to use them.

## Results

### Overview of the Genomic Composition of *S. enterica* Isolates

A summary of the genomic characterization of 19 *S. enterica* isolates included in this study is shown in Table 2. The genomes ranged in size from 4.65 to 5.03 Mbp and GC contents were between 52.1 and 52.2%. The number of coding DNA sequences genes (CDSs) ranged from 4,623 in the smallest genome to 5,150 in the largest genome. The number of *tRNA* genes ranged from 78 up to 95 genes. The quantity of CDS and *tRNA* typically varied depending on the presence or absence of plasmids, prophage insertions or transposons.

**TABLE 2:** Genomic characterization of *S. enterica* isolates used in this study.

**Isolate name**	**PT**	**Genome length (bp)**	**Contigs**	**GC content (%)**	**CDS**	**tRNA**	**Plasmids**
***S.* Typhimurium**							
Sal-12	44	5030924	75	52.1	5150	90	1
Sal-43	135	5016036	91	52.1	5116	90	1
Sal-240	170	4847821	89	52.1	4891	90	0
Sal-1000	135	4898507	71	52.2	4964	90	1
Sal-1003	170	4848064	97	52.1	4882	92	0
Sal-1013	9	4899119	85	52.2	4984	86	1
Sal-1576	135a	4909277	87	52.2	5003	92	1
Sal-1657	135	4903776	87	52.2	4991	84	1
Sal-1705	135	4894790	77	52.2	4952	95	1
Sal-2103	135a	4955897	88	52.2	5052	88	1
Sal-2323	9	4894811	91	52.2	4975	84	1
Sal-2327	135a	4981981	82	52.1	5062	80	2
Sal-2355	135	4885263	76	52.2	4962	95	1
Sal-2413	108	4914103	85	52.1	5010	89	1
***S.* Infantis**							
Sal-576		4649565	65	52.3	4623	83	0
Sal-1023		4661356	56	52.3	4663	78	1
Sal-1457		4803534	64	52.2	4881	89	1
***S.* Singapore**							
Sal-1234		4721851	50	52.2	4772	81	0
Sal-2350		4704169	60	52.2	4714	86	0

**FIGURE 1:**
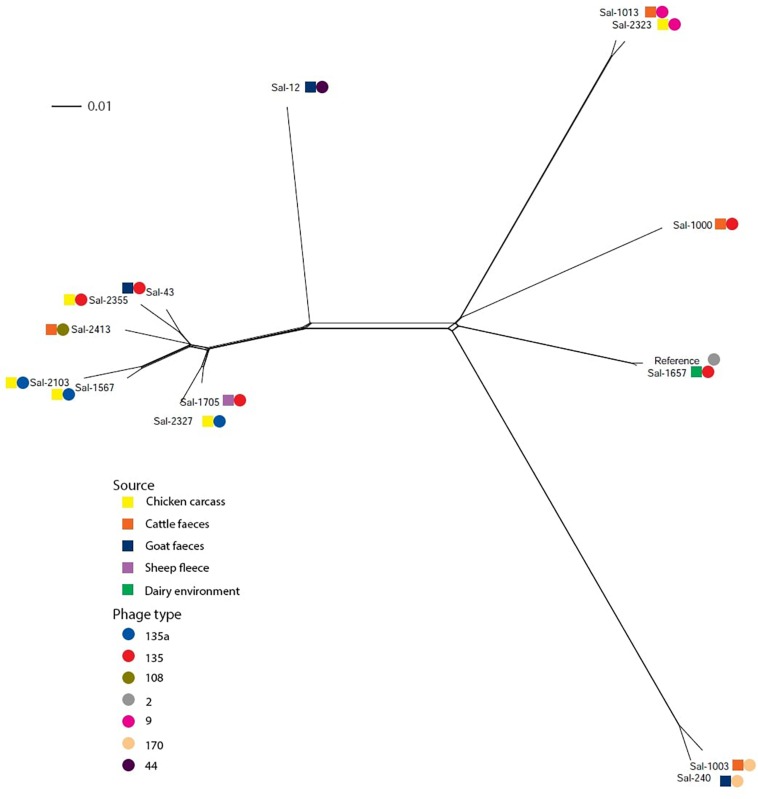
Phylogenic networks of *S.* Typhimurium genomes constructed using the Neighbor-Joining algorithm, Neighbor-Net, in SplitsTree4. Reference refers to *S.* Typhimurium LT2 reference genome (Accession Number: AE006468). Colored squares represent the isolation source and the colored circle represent the phage type. The scale bar distance represents 0.01 nucleotide substitutions per site.

Variant calling among the *S.* Typhimurium isolates was performed by mapping raw sequence reads with *S.* Typhimurium LT2 (NC_003197.2) using Snippy. The core SNP file identified 26,400 variable sites. Using the NeighborNet algorithm (Bryant and Moulton, 2004) in the SplitsTree4 package (Huson, 1998), a phylogenic network was constructed from the core SNP file. The phylogenic network of *S.* Typhimurium isolates in this study is shown in Figure 1. Six of eight *S.* Typhimurium PT135/PT135a isolates formed one clonal group, which also included the PT108 isolate. Other PT135 isolates were more divergent, including Sal-1657 which clustered near the *S.* Typhimurium LT2 reference genome. This strain is the only PT135/135a isolate recovered from a dairy factory. Two other clusters were evident, one of which included both PT170 strains, isolated from cattle feces and goat feces in 2002 and 2001, respectively. The other highly clonal group included both PT9 isolates.

### Characterization of Mobile Genetic Elements of *S. enterica* Isolates

#### Characterization of Plasmids Carried by *S. enterica* in This Study

**FIGURE 2:**
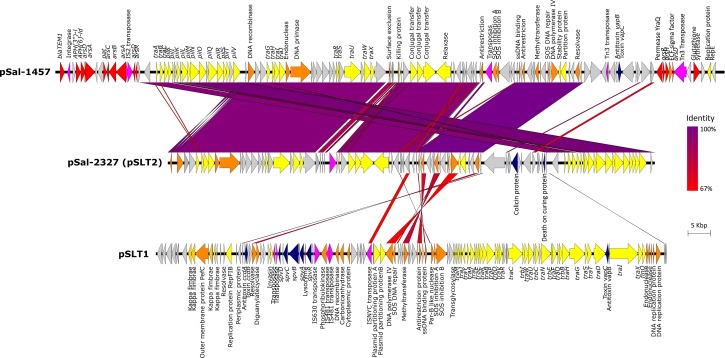
The alignment of three plasmids, pSal-1457, pSal-2327, and pSLT1 using Easyfig. Gene annotations are characterized by function as follows: resistance genes (red), conjugal transfer associated genes (yellow), virulence genes (blue), DNA replication (orange), transposons/integrases (pink), and hypothetical proteins (gray).

Analysis of WGS data using Plasmid Finder identified three types of plasmid profiles, as represented in Figure 2. Twelve of 14 *S.* Typhimurium isolates carried a ∼94 kbp IncF virulence plasmid. The plasmid contains three virulence operons, the *spv* virulence loci which including five genes *spvRABCD*, the *ccdAB* antitoxin/toxin loci and the *vapBC* antitoxin/toxin loci all of which were conserved in all genomes. It also carries a chromosome-independent *fer* fimbrial operon. When analyzed in the NBCI BLASTn platform, the plasmid sequence shared 99% sequence identity with plasmid pSLT plasmid carried by *S.* Typhimurium LT2 strain (ATCC^®^ 14028^TM^, Accession Number: NC_003277). Multiple alignment of the 12 pSLT plasmid sequences extracted from genomes of isolates in this study identified 99.96% sequence homology with a total of 106 SNPs. Non-synonymous variances include a SNP leading to amino acid modification Gln216Leu in kappa-fimbrae chaperone gene *pefD* in isolate Sal-2323 and a SNP leading to AA change Trp141Cys and in the plasmid SOS inhibition protein of isolate Sal-2355. Additionally, two isolates of phage type PT135A, Sal-2103, and Sal-1576, possess an identical non-synonymous SNP in residue 26 of the *ccdB* toxin gene causing AA modification of aspartic acid (Asp) to valine (Val). The plasmid did not carry any identified antibiotic, heavy metal or biocide resistance genes.

Only one isolate carried more than one plasmid. In addition to pSLT described above, Sal-2327 also possessed an additional IncI1 incompatibility group virulence plasmid 89.6 kbp in length. With the exception of the presence of a-DNA adenine methyltransferase and a small number of DNA replication proteins, this plasmid shares very little homology with pSLT1. However, BLASTn comparison showed 85.7% sequence identity to the *S.* Typhimurium pSLT2 plasmid (Accession Number: KF290378). While this plasmid did contain complete plasmid replication and transfer coding machinery, it did not contain any known virulence or antimicrobial resistance gene cassettes.

A third plasmid was identified in *S.* Infantis strain Sal-1457 which was isolated from a chicken carcass in 2005. This plasmid was 121.4 Kbp in length and contained a *vapBC* toxin/antitoxin loci and a number of antimicrobial resistance operons. The plasmid included a Class 1 integron containing an AMR gene cassette: integrase (*intI1*), aminoglycoside 3-phosphotransferase [*aph(3^″^)-I*], aminoglycoside 6-phosphotransferase [*aph(6^″^)-ld*], b-lactamase (*bla_*TEM*–1_*) and a trimethoprim resistance conferring dihydrogen reductase (*dfrA5*). Another feature of this plasmid is the presence of two separate transposons both of which harbored arsenic resistance operons. The first transposon included a Tn*5393* transferase flanking an *arsRHD* operon, and the second containing two tandem IS2-family transposases followed by an *arsABCD* operon.

Plasmids were not identified in *S.* Typhimurium isolates of phage type PT170, both *S.* Singapore isolates, and two of three *S.* Infantis isolates.

#### Phage Insert Regions and Their Antimicrobial Resistance Determinants

**FIGURE 3:**
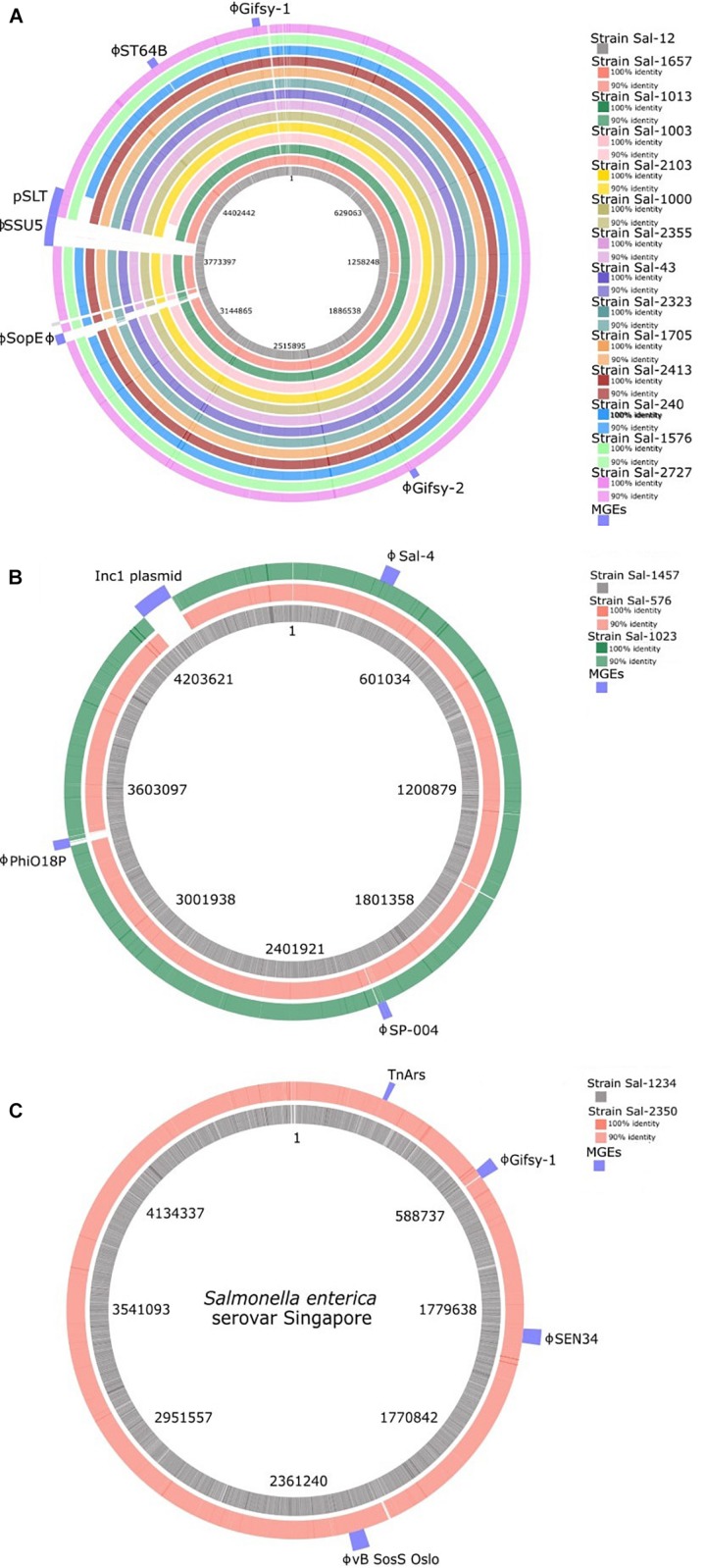
BLAST ring visualization of genomes of **(A)** *S.* Typhimurium, **(B)** *S.* Infantis, and **(C)** *S.* Singapore isolates analyzed in this study. The location of mobile genetic elements is indicated in the outer ring. Mobile genetic elements consist of the majority of intra-serotype variation between isolates. ϕ, complete prophage.

Prophage detection using the online platform PHASTER identified 70 complete prophage insertions among the 19 *S. enterica* isolates in this study. Among the phage insertion sites, fourteen prophage types were identified (Table 3). Phage inserts accounted for the majority of genomic diversity between strains (Figure 3). All genomes contained between two to four prophage sequences with the exception of Sal-12 which harbored five different prophage insertions. The Lambda-like prophage insertions Gifsy-1, Gifsy-2, and ST64B were highly conserved among *S.* Typhimurium isolates. Prophage ST64B, a P22 family of prophage was complete in all *S.* Typhimurium isolates with the exception of two PT170 isolates, Sal-240 and Sal-1003, which were missing a phage antitermination protein, DNA invertase, phage holin, an endopeptidase *rzpD* and two hypothetical phage proteins of unknown function. Gifsy-2 prophage contains two virulence factors, superoxide dismutase (Cu and Zn) encoded by the *sodC* gene, and *msgA*, a virulence gene of unknown function. A Gifsy-2 phage was also identified in both *S.* Singapore isolates which also contains a *spvRABC* virulence operon.

**TABLE 3:** Prophage sequences identified in isolates in this study.

**Isolate name**	**PT**	**Gifsy-1**	**Gifsy-2**	**ST64B**	**SPN9CC**	**HP2**	**PhiO18P**	**RE-2010**	**Sal-4**	**SEN34**	**SopEφ**	**vB SosS Oslo**	**SP-004**	**SSU5**
***S.* Typhimurium**														
Sal-12	44	+	+	+	–	–	–	–	–	–	+	–	–	+
Sal-43	135	+	+	+	–	+	–	–	–	–	–	–	–	–
Sal-240	170	+	+	–	+	+	–	–	–	–	–	–	–	–
Sal-1000	135	+	+	+	–	–	–	–	–	–	–	–	–	–
Sal-1003	170	+	+	–	+	+	–	–	–	–	–	–	–	–
Sal-1013	9	+	+	+	–	–	–	+	–	–	–	–	–	–
Sal-1576	135a	+	+	+	–	–	–	–	–	–	–	–	–	–
Sal-1657	135	+	+	+	–	–	–	–	–	–	–	–	–	–
Sal-1705	135	+	+	+	–	–	–	–	–	–	–	–	–	–
Sal-2103	135a	+	+	+	–	+	–	–	–	–	–	–	–	–
Sal-2323	9	+	+	+	–	+	–	–	–	–	–	–	–	–
Sal-2327	135a	+	+	+	–	–	–	–	–	–	–	–	–	–
Sal-2355	135	+	+	+	–	–	–	–	–	–	–	–	–	–
Sal-2413	108	+	+	+	+	–	–	–	–	–	–	–	–	–
***S.* Infantis**														
Sal-576		–	–	–	–	–	–	–	+	–	–	–	+	–
Sal-1023		–	–	–	–	–	+	–	+	–	–	–	+	–
Sal-1457		–	–	–	–	–	–	+	+	–	–	–	+	–
***S.* Singapore**														
Sal-1234		+	–	–	–	–	–	–	–	+	–	+	–	–
Sal-2350		+	–	–	–	–	–	–	–	+	–	+	–	–

Sal-12 is the only isolate to carry SopEΦ, a P2-like phage closely related to Fels-2 found in LT2. This phage harbors virulence factor gene *sopE*. Phage SPN9CC (JF900176) a P22-like phage was identified in only three isolates: PT170 strains Sal-240, Sal-1003, and PT135 strain Sal-2413. Sal-1013 and Sal-2323, both phage type 9, carry a phage that shares 99.9% homology with *Salmonella* phage RE-2010.

#### Transposon-Conferred Resistance Genes

The nucleotide sequence of the two *S.* Singapore isolates examined in this study included a gene cluster containing an arsenic resistance operon. This included two integrase genes flanked by an arsenic resistance transcriptional repressor (*arsR*), arsenate reductase (*arsC*), arsenic resistance transacting repressor (*arsD*), arsenical pump-driving ATP-ase (*arsA*), and arsenical-resistance protein (*arsB*). The 5,166 bp transposon containing the *arsRCDAB* operon shared 100% homology with an arsenic resistance gene cassette identified in *S.* Thompson strain (ATCC^®^ 8391^TM^, Accession Number: CP011396) and 99% query identity with *S.* Kentucky strain CVM29188 (Accession Number: ABAK02000001.1). The genome of *S.* Typhimurium isolates Sal-2103 contained an IS630-family transposon integrated into a Type IV secretion system. The transposon contains a tetracycline resistance gene cassette, inclusive of a tetracycline resistance regulatory gene *tetR*, tetracycline resistance efflux pump encoded *tetA*, and an EamA family multidrug/metabolite transporter flanked by two IS630-family transposases.

### Characterization of Chromosomally Located Antimicrobial Resistance Determinants

Among all 19 isolates, there were no mutations identified in the quinolone resistance determining region of DNA topoisomerase genes *gyrB*, *parC*, and *parE*. Additionally, there were no isolates found to carry quinolone resistance genes *qnrA*, *qnrB*, or *qnrS*. An isolated SNP was identified in the *gyrA* gene of nalidixic acid-resistant isolate Sal-1657 in the 87th codon leading to the amino acid substitution aspartate (Asp) to glycine (Gly). Analysis of chromosomal DNA and mobile genetic elements found that there was little difference in the gene content that confers resistance to biocides that could account for any differentiation in the biocide resistance phenotype of isolates in this study. With the exception of the *tetAR* genotype identified in Sal-2103 there were no tetracycline genes (*tetABCDGR*) found in any isolate in this study.

### Antimicrobial Susceptibility Profile of Isolates in This Study

The antibiotic resistance profiles of all 19 *S. enterica* isolates are shown in Table 4. Antibiotic susceptibility testing identified that 7 isolates were resistant to at least one antibiotic. All isolates (100%) were susceptible to chloramphenicol, ciprofloxacin, florfenicol, sulfamethoxazole-trimethoprim, gentamycin, and kanamycin. Isolate Sal-1457, which was found to harbor a plasmid-borne *bla_*TEM*–1_* gene and *strAB* gene in a Class 1 integron, was found to be resistant to ampicillin, streptomycin and cefoxitin. All other isolates were susceptible to the β-lactam class of antibiotics with the exception of Sal-576 and Sal-1023 (both *S.* Infantis) that were resistant to cefoxitin. Isolate Sal-2103 was resistant to tetracycline, consistent with its transposon-carrying *tetAR* genotype. Isolate-1657, which possessed a mutation to DNA gyrase subunit gene *gyrA*, was resistant to nalidixic acid but not resistant to ciprofloxacin, also a fluoroquinolone antibiotic. Despite not having any antibiotic resistance genes known to confer sulphafurazole resistance (*sul1*, *sul2*, or *sul3*), isolates Sal-43 and Sal-1576 were found to be resistant to this antibiotic. Similarly, all three *S.* Infantis isolates are resistant to cefoxitin, however no resistance genes associated with resistance to this antibiotic were detected in the WGS data of these isolates when analyzed against the database libraries.

**TABLE 4:** The antibiotic resistant phenotype of *S. enterica* isolates in this study.

**Isolate name**	**PT**	**Genotype**	**AMP**	**ST**	**TE**	**SF**	**FOX**	**FFC**	**NOR**	**K**	**CN**	**CIP**	**C**	**SXT**	**NA**
***S.* Typhimurium**															
Sal-12	44	–	S	S	S	S	S	S	S	S	S	S	S	S	S
Sal-43	135	–	S	S	S	R	S	S	S	S	S	S	S	S	S
Sal-240	170	–	S	S	S	S	S	S	S	S	S	S	S	S	S
Sal-1000	135	–	S	S	S	S	S	S	S	S	S	S	S	S	S
Sal-1003	170	–	S	S	S	S	S	S	S	S	S	S	S	S	S
Sal-1013	9	–	S	S	S	S	S	S	S	S	S	S	S	S	S
Sal-1576	135a	–	S	S	S	R	S	S	S	S	S	S	S	S	S
Sal-1657	135	Δ*gyrA*	S	S	S	S	S	S	S	S	S	S	S	S	R
Sal-1705	135	–	S	S	S	S	S	S	S	S	S	S	S	S	S
Sal-2103	135a	*tetA*	S	S	R	S	S	S	S	S	S	S	S	S	S
Sal-2323	9	–	S	S	S	S	S	S	S	S	S	S	S	S	S
Sal-2327	135a	–	S	S	S	S	S	S	S	S	S	S	S	S	S
Sal-2355	135	–	S	S	S	S	S	S	S	S	S	S	S	S	S
Sal-2413	108	–	S	S	S	S	S	S	S	S	S	S	S	S	S
***S.* Infantis**															
Sal-567		–	S	S	S	S	R	S	S	S	S	S	S	S	S
Sal-1023		–	S	S	S	S	R	S	S	S	S	S	S	S	S
Sal-1457		*bla*_*TEM–1*_, *strAB*	R	R	S	S	R	S	S	S	S	S	S	S	S
***S.* Singapore**															
Sal-1234		–	S	S	S	S	S	S	S	S	S	S	S	S	S
Sal-2350		–	S	S	S	S	S	S	S	S	S	S	S	S	S
**Control**															
25922			S	S	S	S	S	S	S	S	S	S	S	S	S

*NB: AMP, ampicillin; P, penicillin; ST, streptomycin; TE, tetracycline; SF, sulphafurazole; FOX, cefoxitin; FFC, florfenicol; NOR, norfloxacin; K, kanamycin; E; erythromycin; CN, gentamicin; CIP, ciprofloxacin; C, chloramphenicol; SXT, trimethoprim-sulfamethoxazole; NA, nalidixic acid; S, susceptible; R, resistant.*

### Heavy Metal and Disinfectant Susceptibility of Isolates in This Study

Heavy metal susceptibility testing of the 19 *Salmonella* isolates was performed against arsenic, lead, cadmium, copper, cobalt, and zinc (Table 5). Isolates Sal-1003, Sal-1023, Sal-1234, and Sal-2350, all of which possess arsenic resistance gene operons, were found to be viable at 30 mM NaAsO_2_, the highest concentration tested in this study. This concentration is more than 5× the MBC of the other 15 isolates at 6 mM NaAsO_2_. The MIC of all isolates to Pb(NO_3_)_2_ was recorded as 10 mM. However, viable cells were found at the highest concentration tested of 15 mM (MBC not determined). A concentration of 0.5 mM of CdCl_2_ was bactericidal to all but three isolates. Isolates Sal-1013, Sal-1657, Sal-2355 were viable at 0.5 mM but showed no visible growth at this concentration (inhibited). Isolate Sal-1705 showed visible growth and viability at 0.5 mM but was inhibited at 1.5 mM. A concentration of 1.5 mM of CdCl_2_ was bactericidal to all isolates. There were no differences in the MIC or MBC of all isolates utilized in this study when exposed to CoCl_2_, CuSO_4_, and ZnCl_2_.

**TABLE 5:** Heavy metal and disinfectant sensitivities of *S. enterica* isolates in this study.

**Bacterial strains**	**As^3+^**		**Pb^2+^**		**Cd^2+^**		**Cu^2+^**		**Co^2+^**		**Zn^2+^**		**Tri**		**BZ**		**CHL**	
	**MIC^a^**	**MBC^b^**	**MIC**	**MBC**	**MIC**	**MBC**	**MIC**	**MBC**	**MIC**	**MBC**	**MIC**	**MBC**	**MIC**	**MBC**	**MIC**	**MBC**	**MIC**	**MBC**
**S. Typhimurium**																		
Sal-12	6	6	10	> 15	0.5	0.5	8	8	4	4	4	4	0.12	1	16	16	4	32
Sal-43	6	6	10	> 15	0.5	0.5	8	8	4	4	4	4	0.12	1	16	16	2	16
Sal-240	6	6	10	> 15	0.5	0.5	8	8	4	4	4	4	0.12	2	16	16	2	32
Sal-1000	6	6	10	> 15	0.5	0.5	8	8	4	4	4	4	0.12	0.5	16	16	4	32
Sal-1003	10	> 30	10	> 15	0.5	0.5	8	8	4	4	4	4	0.25	1	16	16	2	16
Sal-1013	6	6	10	> 15	0.5	1.5	8	8	4	4	4	4	0.25	2	16	16	4	16
Sal-1576	6	6	10	> 15	0.5	0.5	8	8	4	4	4	4	0.12	2	16	16	2	4
Sal-1657	6	6	10	> 15	0.5	1.5	8	8	4	4	4	4	0.25	2	16	16	8	64
Sal-1705	6	6	10	> 15	1.5	1.5	8	8	4	4	4	4	0.12	1	16	16	8	32
Sal-2103	6	6	10	> 15	0.5	0.5	8	8	4	4	4	4	0.12	1	16	16	8	32
Sal-2323	6	6	10	> 15	0.5	0.5	8	8	4	4	4	4	0.12	1	16	16	4	16
Sal-2327	6	6	10	> 15	0.5	0.5	8	8	4	4	4	4	0.12	2	16	16	8	16
Sal-2355	6	6	10	> 15	0.5	1.5	8	8	4	4	4	4	0.25	2	16	16	4	16
Sal-2413	6	6	10	> 15	0.5	0.5	8	8	4	4	4	4	0.12	1	16	16	8	32
**S. Infantis**																		
Sal-576	6	6	10	> 15	0.5	0.5	8	8	4	4	4	4	0.12	0.12	16	16	2	4
Sal-1023	10	> 30	10	> 15	0.5	0.5	8	8	4	4	4	4	0.12	2	16	16	2	8
Sal-1457	6	6	10	> 15	0.5	0.5	8	8	4	4	4	4	0.12	2	16	16	2	8
**S. Singapore**																		
Sal-1234	10	> 30	10	> 15	0.5	0.5	8	8	4	4	4	4	0.12	2	16	16	2	16
Sal-2350	10	> 30	10	> 15	0.5	0.5	8	8	4	4	4	4	0.25	1	16	16	4	16
**Control**																		
25922	6	6	10	> 15	0.5	0.5	8	8	4	4	4	4	0.12	1	16	16	4	8

*MIC, minimum inhibitory concentration; MBC, minimum bactericidal concentration; TRI, triclosan; BZ, benzalkonium chloride; CHL, chlorhexidine. ^*a*,*b*^Units in mg/L.*

Overall, all isolates had the same (16 μg/ml) MIC and MBC to benzalkonium chloride. While the MIC for triclosan ranged from 0.125 to 0.25 μg/ml, the MBC was more varied from 0.125 to 2 μg/ml with *S.* Infantis and *S.* Typhimurium ST135 isolates dominating the higher MBC concentration range. There was a marked difference between the MIC and MBC of chlorhexidine. MIC values ranged from 2 to 8 μg/ml and MBC values from 4 to 64 μg/ml. Sal-1657 recorded the highest MBC value, which was a 16-fold increase on the lowest MBC value recorded (Sal-576).

## Discussion

This study utilized WGS and comparative genomics to characterize the resistance genes present in the genomes of 19 *S. enterica* strains isolated in Australia and determine the location of these genes within the chromosome and mobile genetic elements. Traditional phenotypic susceptibility testing was also performed to evaluate prediction of phenotypic traits from genotypic data, and determine any associated discrepancies. The use of WGS may reduce the rate of discrepancies caused by unstandardized approaches to phenotypic susceptibility testing and may also identify resistance markers not routinely screened for in phenotypic analysis (Zankari et al., 2013). WGS has the capacity to circumvent the limitations of traditional screening methods, allowing for the detection of AMR genes and their location within the associated bacterial genome, and identify mobile genetic elements which may lead to dissemination of AMR genes throughout the food production chain and clinical settings. Due to the high reliability, reproducibility and availability of WGS technology, this methodology is fast becoming the gold standard for molecular analysis (Zankari et al., 2013; Portmann et al., 2018). The potential application of WGS technologies for the detection of antibiotic susceptibility phenotypes has previously been demonstrated in *E. coli* and *Klebsiella pneumoniae*, where genome-based prediction yielded high specificity values of 96 and 97% (Stoesser et al., 2013). Similarly, quantitative correlations between *Salmonella* isolates studied in the United States with a resistant phenotype and a resistant genotype were identified in 99% of cases (McDermott et al., 2016). A recent study concerning *S. enterica* found that AMR phenotype and genotype profiles of 3,491 isolates were strongly correlated, with 97.82% in agreement (Neuert et al., 2018). This current study characterized the antibiotic, heavy metal and disinfectant resistance genotype and mobile genetic elements of 19 *Salmonella* enterica isolates using WGS data. Whilst this current study did not have a sufficient sample size to infer the statistical significance of a correlation, it could be elucidated that antibiotic resistance genotype profiles were correct, in the most part, in predicting the resistance phenotype. Bioinformatic analysis of WGS was able to detect tetracycline, penicillin, ampicillin and nalidixic acid resistant phenotypes in this study. However, sulphafurazole and cefoxitin resistance could not be predicted using the methodology employed. There is likely mechanisms currently unknown, or not included in the database libraries. Thus the mechanism(s) remain unknown in this case.

In addition to its utility in phenotypic prediction through genotypic interrogation, SNP subtyping shows promising application in epidemiological surveillance and outbreak investigations (Ford et al., 2018). In this study, the application of SNP subtyping could differentiate all isolates from one another, including those that shared a similar phage type. Interestingly, clustering did not group PT135 and PT135a in distinct subgroups. Separation of PT135 and PT135a through phage typing has been used in Australia to provide increased resolution in epidemiological investigations (McPherson et al., 2006).

A number of cellular pathways used by *Salmonella* to resist disinfectants have been previously identified. Resistance to quaternary ammonium compounds (QACs), as described in previous studies, can by conferred by the acquisition of transmembrane efflux pump genes including *qacA*, *qacB*, *qacC*, *qacH*, *smr*, and *qacΔE1*, the latter of which is conserved in the Class 1 integron (Deng et al., 2017). This study did not detect known QAC or triclosan resistance determinants in the genome sequences of the 19 isolates including in this study, which is supported by the phenotypic data. Although chlorhexidine MICs were within a small range (2–8 mg/L), MBC values showed higher variation (4–64 mg/L). The reasons for this disparity were not identified in this study and require further investigation. Arsenic resistance operons were detected in five isolates. Although four of these showed a higher MIC to arsenic, one isolate (Sal-1457) which possessed two plasmid-encoded arsenic resistance operons, did not demonstrate increased tolerance *in vitro*. Research in the area of biocide and heavy metal resistance is lacking, with much of the focus on validating WGS as an antibiotic resistance detection tool due to the clinical implications of AMR. Consequently, there are few bioinformatics tools available that have been validated that can detect biocide and heavy metal resistance in bacterial pathogens.

Antibiotic resistance presents a significant issue for public health and clinical outcomes for patients (Krueger et al., 2014). In response, the World Health Organization has identified antibiotic resistant *Salmonella* as a species of global public health significance and a priority for future research and development strategies (WHO, 2017). There have been a number of emerging reports of antibiotic resistant *Salmonella* isolated from Australian food production chains. Among 217 isolates from Australian beef cattle, a resistant phenotype was observed in the presence of ampicillin (7.5%), streptomycin (7.5%), tetracycline (6.6%), and trimethoprim-sulfamethoxazole (7.5%, Barlow et al., 2015). However, this prevalence is relatively low when compared to *Salmonella* AMR data from the United States, with a previous study reporting higher resistance rates to ampicillin (9.2%), streptomycin (15.5%), tetracycline (66.9%), and trimethoprim-sulfamethoxazole (14.9%) among cattle isolates in Food and Drug Administration [FDA] (2015). The results of the current study show similar trends to previous work (Barlow et al., 2015), and suggest that the overall prevalence of clinically relevant AMR in Australian *Salmonella* isolated from the environment is relatively low, with 7 out of 19 isolates demonstrating resistance to only one antibiotic and only one isolate with a MDR profile.

This study reported relatively high sensitivity to zinc (4 mM), copper (8 mM), and cobalt (4 mM) in all isolates. Previous studies have reported copper tolerance up to 24 mM and zinc tolerance at 8 mM in *Salmonella* isolated from swine feed and feces and the carriage of the copper, zinc cadmium efflux pump gene *czcD* was significant associated with increased MIC (Medardus et al., 2014). Tetracycline, nalidixic acid, and ampicillin are early generation antibiotics and have been used extensively in food producing animals (Silbergeld et al., 2008). This may have contributed to the representation of the resistance genotype/phenotype present in this study. Resistance to nalidixic acid was identified in one isolate in this study that possessed a SNP in the QRDR of DNA gyrase gene *gyrA* resulting in amino acid change Asp87Gly. Mutations to the Quinolone Resistance Determining Regions (QRDR) in DNA gyrase genes, *gyrA* and *gyrB*, and DNA Topoisomerase genes, *parC* and *parE*, have been detected in fluoroquinolone-resistant strains of *Salmonella*, including those resistant to ciprofloxacin, norfloxacin and nalidixic acid (Gal-Mor et al., 2010; Utrarachkij et al., 2017; Vital et al., 2017). Nalidixic acid is not currently used as a treatment option of salmonellosis in Australia, with amoxicillin, trimethoprim-sulfamethoxazole and azithromycin used in human cases and apromycin used in calves, pigs and broiler infections (Lean et al., 2013).

It is interesting to note that the majority of resistance determinants identified in this study were present on transposable elements, either on plasmids or chromosomally located MGEs. All but six strains harbored large conjugative plasmids, with an InclI plasmid carried by Sal-1457 conferring resistance to ampicillin and streptomycin, found on a class 1 operon. This plasmid also carried two transposons flanking arsenic resistance gene cassettes. Additionally, Sal-2103 carries a transposon that confers resistance to tetracycline while transposons carried by Sal-2350 and Sal-1234 conferred arsenic resistance. The presence of transposons and mobile genetic elements nested within larger mobile genetic elements, such as plasmids or phage, has been found to promote faster dissemination of AMR genes (Han et al., 2012; Sheppard et al., 2016). Plasmids analyzed in this study also harbored a number of virulence and toxin/antitoxin genes including the *spv* operon that is associated with survival in macrophages through actin depolarisation and host cell apoptosis (Guiney and Fierer, 2011). The superoxide dismutase encoding gene was also identified in two different prophage types, including Gifsy-2 found in all *S.* Typhimurium isolates. SodCI enzyme mutants have been found to possess reduced survivability to macrophage oxidative burst, through the inability to convert phagocytic superoxide to peroxide (Ammendola et al., 2008). A 38.5 kbp prophage (SopEφ) found in *S.* Typhimurium isolate Sal-12 contained the *sopE* gene, a TIII-secreted protein that enables the internalization of *Salmonella* into the host cell (Vonaesch et al., 2014). Whilst no AMR genes were present in these prophage, these findings suggest the role of these MGEs in the dissemination MDR resistance phenotypes in *Salmonella*.

## Conclusion

Antibiotic resistance is an increasing important issue which has serious implications for human and veterinary science. The presented study suggests that there are low levels of clinically relevant antibiotic resistance among *Salmonella* isolated from Australian food-producing animals and that mobile genetic elements such as transposons and plasmids play a central role in the dissemination of AMR genes. Further surveillance in Australia of AMR prevalence among *Salmonella* from food animal-related sources, with an expanded cohort and larger serotype representation, is recommended and this study provides a basis for further investigation.

## Author Contributions

AW completed the experiments, and collected and analyzed the data. AW, NF, EF, and DK conceived and designed the experiments, and wrote, revised, and approved the final version of the manuscript.

## Conflict of Interest Statement

The authors declare that the research was conducted in the absence of any commercial or financial relationships that could be construed as a potential conflict of interest.

## Footnotes

1 http://health.gov.au/internet/fr/publishing.nsf/Content/australia-foodborne-illness-reduction-strategy-2018%E2%80%932021

2 https://github.com/tseemann/snippy

3 http://genomicepidemiology.org

4 http://bacmet.biomedicine.gu.se/

5 www.geneious.com
